# Changes in Lower Limb Axial Alignment, Gait Biomechanics, and Plantar Force in Crowe Type IV Hip Dysplasia After Total Hip Arthroplasty: A Mean Ten‐Year Follow‐Up Retrospective Cohort Study

**DOI:** 10.1111/os.70258

**Published:** 2026-02-06

**Authors:** Huiling Chen, Junqing Wang, Yan Li, Steve T. L. Pambayi, Shijiu Yin, Jing Yang, Yong Nie, Yi Zeng

**Affiliations:** ^1^ Department of Orthopedic Surgery and Orthopedic Research Institute West China Hospital, Sichuan University Chengdu China; ^2^ Beijing Hospital, National Center of Gerontology Institute of Geriatric Medicine, Chinese Academy of Medical Sciences & Peking Union Medical College Beijing China; ^3^ Department of Radiology West China Hospital, West China Medical School, Sichuan University Chengdu China

**Keywords:** Crowe IV DDH, gait analysis, lower limb alignment, plantar force, THA

## Abstract

**Objectives:**

Total hip arthroplasty (THA) is the gold standard for treating Crowe IV developmental dysplasia of the hip (DDH). However, its long‐term effects on lower limb alignment, gait biomechanics, and plantar force in these patients remain underexplored, which is discussed in this article.

**Methods:**

We conducted a retrospective cohort study that included 43 DDH Crowe IV patients who underwent THA between February 2008 and October 2019 and a control group of 43 matched healthy volunteers. Postoperative functional outcomes and quality of life were assessed using the Harris Hip Score, KOOS, AOFAS, and WOMAC scores. Lower limb alignment parameters (MAD, HKA, aTFA, mLDFA, mMPTA, and FO), knee alignment (HMFC, HLFC), and ankle alignment (mLDTA, FACO, and TT) were measured preoperatively, postoperatively, and at follow‐up. Gait analysis and plantar force measurements were performed at the final follow‐up.

**Results:**

With an average follow‐up of 10.2 years, patients showed significant improvement in functional and quality of life scores compared to pre‐surgery. Preoperatively, all patients had knee valgus and ankle varus on the affected side. After THA, most parameters showed reduced valgus alignment, except for HKA and HLFC. On the unaffected side, MAD, aTFA, and HKA indicated preoperative valgus, which was fully corrected post‐THA. Gait analysis revealed restricted lower limb motion and abnormal plantar force distribution that persisted postoperatively in Crowe IV DDH patients.

**Conclusions:**

THA partially corrected abnormal lower limb alignment, gait parameters, and plantar force distribution in DDH Crowe IV patients over long‐term follow‐up.

## Graphical abstract

- This is the first study to examine the global alignment of the hip, knee, ankle, and lower limb after total hip arthroplasty in DDH Crowe type IV patients with a follow‐up period of more than 10 years.
- This is also the first study of its kind (as far as we know) in which both the unaffected and the affected sides were monitored, and full normalization of the alignment of the healthy lower limb was observed after surgery.
- We studied patients' gait biomechanics and plantar force, trying to figure out the functional change after THA.

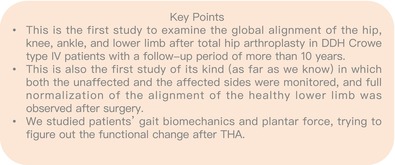

## 1 Introduction

Developmental dysplasia of the hip (DDH) is a common cause of secondary hip osteoarthritis, leading to pain and impaired mobility, greatly impacting functionality and quality of life [1]. DDH is categorized into four types, with Crowe IV being the most severe [2]. Total hip arthroplasty (THA), especially combined with femoral shortening osteotomy (FSO), is the standard approach for Crowe IV DDH patients due to favorable long‐term functional outcomes and acceptable implant survival rates [3]. However, THA in Crowe IV DDH patients can be challenging due to anatomical deformities, limb length discrepancy, and the high risk of potential postoperative complications.

Prolonged high hip dislocation in Crowe IV patients leads to adaptive changes in muscles, soft tissues, and nearby structures, resulting in persistent abnormal lower limb alignment [4, 5]. THA with FSO can restore function and quality of life by reconstructing biomechanical anatomy, but it may introduce new problems due to changes like iliotibial tract stiffness and adaptive walking posture [6]. Recent studies have focused on the effect of THA on correcting lower limb alignment, particularly valgus knee joint alignment in Crowe IV DDH patients [6, 7]. Some studies have shown neutralization of knee and ankle valgus alignment post‐THA, while others have found persistent valgus alignment in the lower limb [8, 9]. Furthermore, the unilateral high hip center has been shown to limit the range of motion in the affected lower limb [10, 11, 12] and compensatory changes in ankle alignment, along with abnormal knee alignment, have been observed but not specifically studied in Crowe IV DDH patients [13, 14, 15]. However, most studies have short follow‐up durations of around 2 years, lacking long‐term investigations beyond 5 years. Moreover, they often neglected changes in the unaffected side and were limited by sample size.

We conducted this 10‐year follow‐up study to address the following scientific questions: (i) to evaluate the long‐term efficacy of THA in improving hip function and patient‐reported outcomes in Crowe IV DDH patients; (ii) to comprehensively assess the postoperative changes in multi‐dimensional biomechanical parameters, including lower limb axial alignment, gait kinematics and kinetics, and plantar force distribution; and (iii) to determine the extent and persistence of any residual biomechanical deviations at a mean follow‐up exceeding 10 years.

## 2 Methods

### 2.1 Ethics

This study was registered in the Chinese Clinical Trial Registry (ChiCTR2200067203), and ethical approval was granted by our Hospital's Clinical Trials and Biomedical Ethics Committee (2022. NO. 1413). Informed consent was obtained from all individual participants included in the study.

### 2.2 Study Design

#### 2.2.1 Patients

**FIGURE 1:**
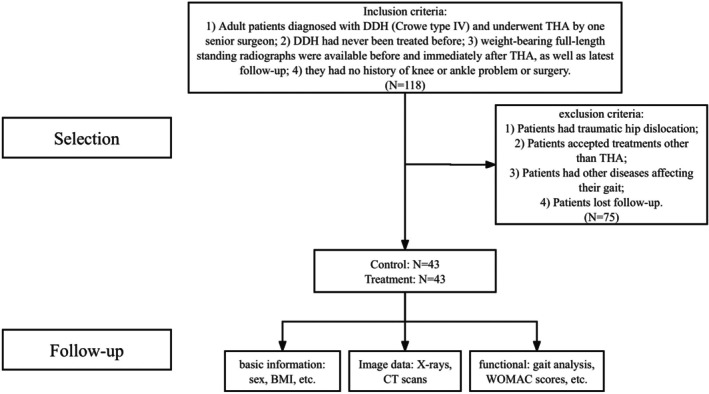
Flow chart shows included patients in this study.

In this retrospective cohort study, 43 patients (64 hips) with Crowe IV DDH who underwent THA from February 2008 to October 2019 were enrolled (Figure 1). Inclusion criteria were as follows: (1) adult patients diagnosed with Crowe IV DDH and treated with THA, (2) no prior DDH treatment, and (3) no history of knee or ankle disease or treatment. Patients with traumatic hip dislocation, musculoskeletal abnormalities, nerve damage, or other gait‐affecting conditions were excluded. For comparison, we selected 43 healthy individuals (86 hips) through a 1:1 matching process considering age, gender, and BMI. DDH patients were sub‐categorized into unilateral and bilateral groups based on their diagnosis, while healthy individuals formed the control group.

#### 2.2.2 Surgical Procedure

All patients underwent THA using the posterolateral approach, performed by five experienced surgeons. The acetabulum cup was implanted in the true acetabulum to restore the anatomic hip's rotation center. Porous‐coated acetabular components (Pinnacle implants, DePuy Orthopedics) were used in all patients. If necessary, a transverse FSO was performed approximately 1–2 cm below the lesser trochanter. A proximally porous‐coated modular stem with a fluted distal element (S‐ROM, DePuy Orthopedics) was implanted.

#### 2.2.3 Postoperative Management

A standardized postoperative protocol was implemented for all patients to minimize potential confounding factors. This included uniform guidelines for pain management, thromboembolic prophylaxis, and wound care. A structured rehabilitation program was initiated, with progressive weight‐bearing and gait training supervised by physiotherapists. Patients were regularly followed in the outpatient clinic to monitor recovery and address any complications promptly.

#### 2.2.4 Clinical Assessment

Clinical evaluations were performed at the final follow‐up visit (mean 10.2 years postoperatively). The Harris hip score (HHS), knee injury and osteoarthritis outcome score (KOOS), and American Orthopedic Foot and Ankle Society (AOFAS) Ankle Hindfoot Scale were measured to assess the function of hip, knee, and ankle joints, respectively. The Western Ontario McMaster Osteoarthritis Index (WOMAC) was applied to assess the overall quality of life for the patients. At these follow‐up examinations, patients were also asked if any other problems had occurred after THA. Postoperative complications were recorded for each patient in detail, including infection, fracture, dislocation, and deep vein thrombosis (DVT). Preoperative scores were retrospectively collected from medical records. While intermediate time points were not systematically captured due to the retrospective nature of this long‐term study, the comparison between the comprehensive preoperative baseline and the long‐term endpoint provides a robust assessment of the sustained impact of THA.

#### 2.2.5 Radiographic Analysis

**FIGURE 2:**
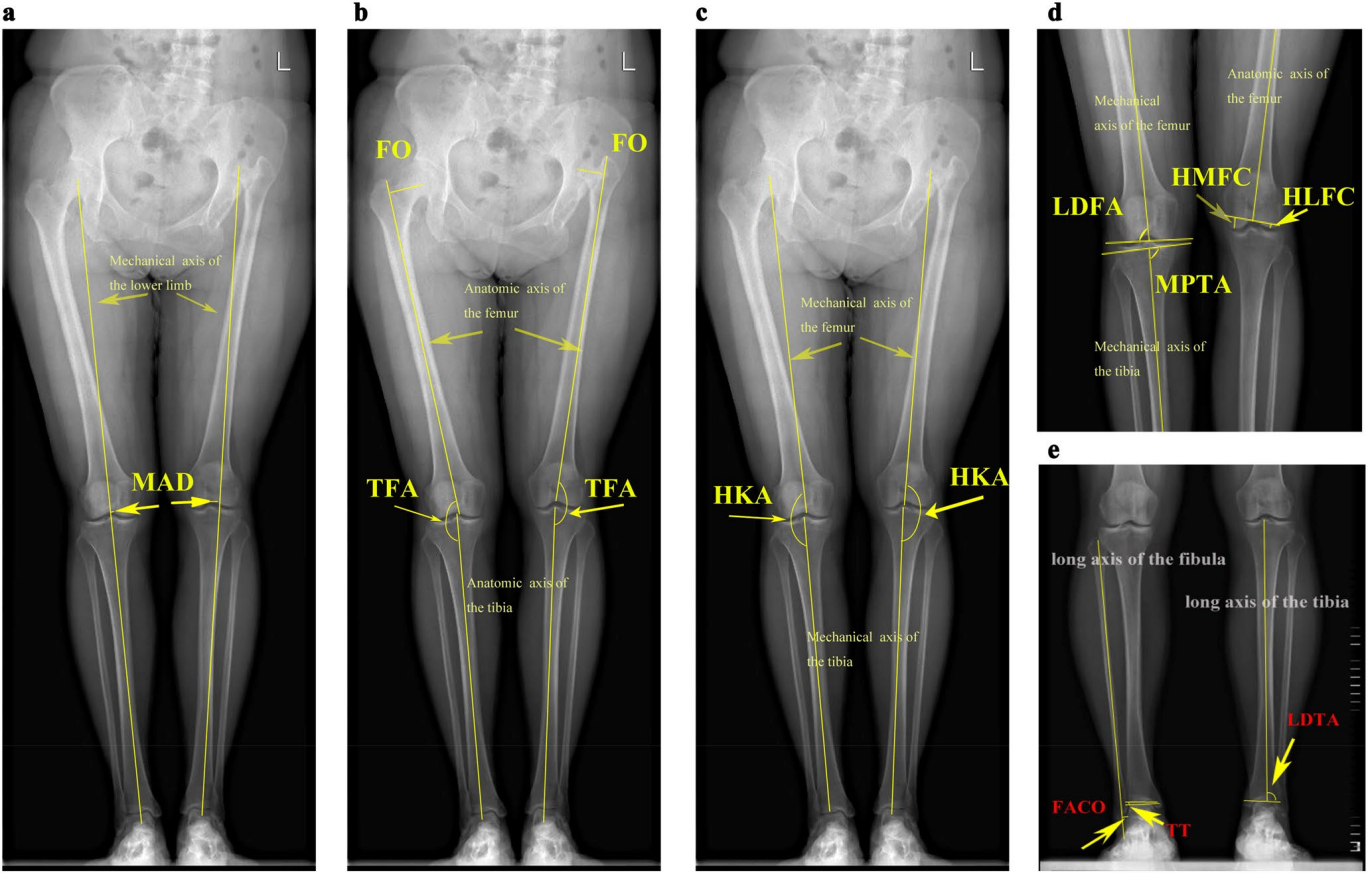
The measurement of radiograph parameters. (a) MAD: The mechanical axis deviation is the distance from the center of the knee to the mechanical axis of the lower limb. (b) FO: The femoral offset is the vertical distance from the center of the femoral head to the center of the femoral canal; TFA: The tibiofemoral angle is the angle formed by the anatomic axis of the femur and the anatomic axis of the tibia. (c) HKA: The hip‐knee‐ankle angle was defined as the angle formed by the mechanical axis of the femur and the mechanical axis of the tibia. (d) LDFA: The mechanical lateral distal femoral angle is the angle formed by the anatomic axis of the femur and the distal femoral joint orientation line; MPTA: The mechanical medial proximal tibial angle is the angle formed by the anatomic axis of the tibia and the proximal tibial joint orientation line; HMFC: The height of medial femoral condyle; HLFC: The height of lateral femoral condyle. (e) FACO: The fibula axis—calcaneal overlap is the greatest distance between the fibula axis and the lateral calcaneal wall; TT: The talar tilt is the angle between the distal tibia articular surfaces and the lines representing the talar dome; LDTA: The mechanical lateral distal tibial angle is the angle between the mechanical axis of the tibia and the distal tibia articular surface.

**FIGURE 3:**
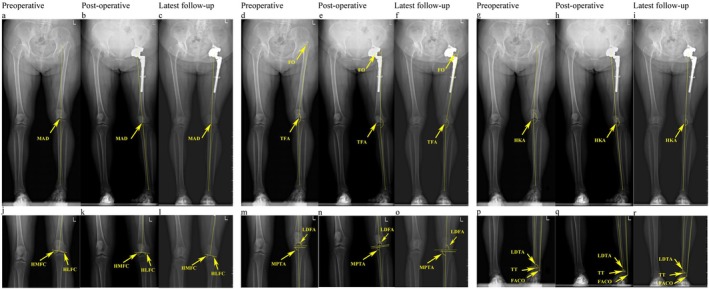
A 62‐year‐old woman was diagnosed with developmental dysplasia of the hip (DDH) Crowe type IV, presenting with a preoperative limb shortening of 5.23 cm. The patient underwent a total hip arthroplasty utilizing a small‐diameter polyethylene‐on‐metal implant with a 40 mm femoral head. A subtrochanteric osteotomy of 4 cm was also performed. The patient was followed for 7.8 years. Follow‐up results indicated the following: (a, d, g, j, m, p) Preoperative measurements of the mechanical axis deviation (MAD), anatomical femoral angle (aTFA), hip‐knee‐ankle angle (HKA), medial proximal tibial angle (mMPTA), hip offset (HMFC), and foot offset (FO) indicated that the affected knee was in a valgus position. In contrast, the femoral angle of the ankle (FACO) and tibial tilt (TT) suggested an ankle varus position. (b, e, h, k, n, q) Immediate postoperative X‐rays demonstrated significant improvements across nearly all indicators related to leg length discrepancy, genu valgus, and ankle varus. (c, f, i, l, o, r) At the final follow‐up, some measurements, including MAD and MPTA, revealed residual valgus positioning of the knee on the affected side.

An anteroposterior view of weight‐bearing full‐length radiographs of lower limbs was obtained to evaluate lower limb alignment (Figure 2). Radiographs were taken at three key time points: preoperatively (as baseline), 1 day after surgery (to assess immediate postoperative alignment), and during the latest follow‐up (mean 10.2 years postoperatively, to evaluate long‐term outcomes). These time points were selected to capture the alignment status at the most critical and clinically relevant phases of care while aligning with the data available in this retrospective cohort study. Each leg was internally rotated to ensure the patella pointed anteriorly (Figure 3). For patients with apparent leg length discrepancy (LLD), radiographs were obtained after placing wooden blocks under the short extremity to ensure the pelvis was level. Leg length discrepancy was defined as the difference in the length of the iliac spine to the ankle joint of both lower limbs. As mentioned in our previous study, we included eight parameters to reflect hip and knee alignments, including the mechanical axis deviation (MAD), the anatomical tibiofemoral angle (aTFA), the hip‐knee‐ankle angle (HKA), the mechanical lateral distal femoral angle (mLDFA), the mechanical medial proximal tibial angle (mMPTA), the vertical height of the medial femoral condyle (HMFC), the vertical height of the lateral femoral condyle (HLFC), and the femoral offset (FO). We also measured the mechanical lateral distal tibial angle (mLDTA), the fibula axis—calcaneal overlap (FACO), and the talar tilt (TT) to determine the ankle alignment and disability [16].

After examination, two authors measured the above parameters independently within the local picture archiving and communication system (PACS) [16, 17]. To ensure the reliability of the radiographic measurements, intra‐ and inter‐observer reliability analyses were performed. Thirty patients were randomly selected. To assess intra‐observer reliability, one observer repeated the measurements after a 1‐month interval. The intraclass correlation coefficient (ICC) using a two‐way random‐effects model for absolute agreement, along with its 95% confidence interval (CI), was calculated according to established benchmarks [18]. The analysis demonstrated that both inter‐ and intra‐observer ICCs spanned from 0.89 to 0.99 (Table S1), indicating highly reproducible measurements.

#### 2.2.6 3D Gait Analysis

The detailed protocol for 3D gait analysis has been described previously [19]. The assessment was conducted at a single time point during the final follow‐up (mean 10.2 years postoperatively) to evaluate the steady‐state gait pattern. Participants walked at a self‐selected pace on a 12‐m walkway while wearing 28 retro‐reflective markers to track the motions of the pelvis, thighs, shanks, and feet. Joint angles and joint moments were calculated using Inverse Kinematics and Inverse Dynamics tools, respectively. A residual reduction algorithm was employed to enhance dynamic consistency. Muscle forces and joint contact forces were estimated using static optimization and JointReaction Analysis tools.

#### 2.2.7 Plantar Forces Analysis

Plantar forces were assessed at the final follow‐up (mean 10.2 years postoperatively) to evaluate long‐term pressure distribution. Measurements were conducted using a 3‐m plantar pressure distribution measurement system (Zebris, Weitnau, Germany), assessing seven zones of the planta pedis: medial heel, lateral heel, midfoot, medial forefoot, intermediate forefoot, lateral forefoot, and toe [20]. The peak force within each region was calculated during walking.

### 2.3 Statistical Analysis

Data were analyzed using IBM SPSS Statistics version 26.0 (IBM, Chicago, IL). Continuous variables are presented as mean and standard deviation or range, depending on their distribution. Categorical variables are reported as percentages and frequencies. The normality of data distribution was confirmed using the Shapiro–Wilk test, and the homogeneity of variance was verified using Levene's test.

Statistical comparisons were structured as follows: (1) For cross‐sectional comparisons between patient groups and healthy controls, one‐way ANOVA was used to compare the affected side of the unilateral DDH group with controls, while Generalized Estimating Equations (GEE) were applied to compare limbs of the bilateral DDH group (accounting for within‐subject correlation between limbs) with controls. (2) For longitudinal analysis within the unilateral DDH group, repeated‐measures ANOVA was employed to compare radiographic and functional parameters across three time points: preoperative, immediately postoperative, and the latest follow‐up. When the assumption of sphericity was violated, the Greenhouse–Geisser correction was applied, and the Welch method was used in cases of heterogeneous variances. Post hoc comparisons were conducted using the LSD and S‐N‐K methods as appropriate.

Survivorship was analyzed with the Kaplan–Meier method, with revision for any reason defined as the endpoint. A two‐sided *p* < 0.05 was considered statistically significant.

## 3 Results

### 3.1 Basic Information

Table 1 shows the baseline characteristics of the study population. For the DDH group, 22 patients received unilateral THA, and 21 received bilateral THA. Fifty‐nine hips (92%) in the DDH group received FSO, and the mean length of osteotomy was 2.72 cm. Preoperative and postoperative LLD were 6.43 cm and 1.33 cm, respectively. No cases with > 2 cm of LLD were identified by the final follow‐up. No significant difference was found in mean age, gender, and BMI between the DDH and control groups. The mean follow‐up period was 10.2 years.

**TABLE 1:** Basic characteristics of participants.

Characteristics	Patient (*N* = 43)	Control (*N* = 43)	*p*
General information			
Age (y), mean (range)	45.5 (22–65)	45.9(25–66)	0.834
Gender, M/F (%)	3/40 (7%/93%)	3/40 (7%/93%)	1
Height (cm), mean ± SD	153.53 ± 6.00	155.40 ± 4.44	0.106
Weight (kg), mean ± SD	54.28 ± 8.01	56.98 ± 5.50	0.072
BMI (kg/m^2^), mean ± SD	23.02 ± 3.02	23.58 ± 1.82	0.299
Affected side, *n* (%)			
Left	16 (37%)	—	
Right	6 (14%)	—	
Bilateral	21 (49%)	—	
Preoperative LLD (cm), mean ± SD	6.43 ± 1.87	—	
Postoperative LLD (cm), mean ± SD	1.33 ± 0.98	—	
Follow‐up duration (y), mean (range)	10.2 (4–12)	—	
Prosthesis information			
Mean size of joint prosthesis (mm)			
Acetabulum	44	—	
Femoral head	28	—	
Friction interface, *n* (%)			
Metal on polyethylene	13 (20%)	—	
Metal on metal	2 (3%)	—	
Metal on ceramic	2 (3%)	—	
Ceramic on polyethylene	2 (3%)	—	
Ceramic on ceramic	45 (71%)	—	
Surgery information			
Operation duration (min), mean ± SD	170.63 ± 52.73	—	
FSO, *n* (%)	59 (92%)		
Length of osteotomy (cm), mean ± SD	2.72 ± 1.14	—	

Abbreviations: FSO, femoral shortening osteotomy; LLD, leg length discrepancy.

### 3.2 Clinical Outcomes

Table 2 shows the results of the HHS, KOOS, AOFAS, and WOMAC scores. At the end of follow‐up, the HHS, KOOS, AOFAS, and WOMAC scores had substantially increased compared with the preoperative status (*p* < 0.001), except for the WOMAC score of the unilateral group (*p* = 0.716).

**TABLE 2:** Clinical outcomes of the enrolled patients.

Characteristics		Values		*p*
		Preoperative	Last follow‐up	
Harris hip score, (mean ± SD)	Bilateral	37.14 ± 11.36	84.52 ± 7.79	***p***_**1**_ **< 0.001**
	Unilateral	43.64 ± 11.15	89.36 ± 4.63	***p***_**2**_ **< 0.001**
	*p*	*p*_3_ = 0.135	***p***_**3**_ **= 0.036**	
KOOS (mean ± SD)	Bilateral	59.29 ± 16.64	16.38 ± 21.10	***p***_**1**_ **< 0.001**
	Unilateral	46.64 ± 20.35	13.55 ± 14.79	***p***_**2**_ **< 0.001**
	*p*	*p*_3_ = 0.094	*p*_3_ = 0.662	
AOFAS Ankle Hindfoot Scale score (mean ± SD)	Bilateral	68.67 ± 7.97	93.90 ± 6.60	***p***_**1**_ **< 0.001**
	Unilateral	64.64 ± 8.95	95.73 ± 3.90	***p***_**2**_ **< 0.001**
	*p*	*p*_3_ = 0.335	*p*_3_ = 0.409	
WOMAC score (mean ± SD)	Bilateral	22.55 ± 1.06	3.57 ± 8.29	***p***_**1**_ **< 0.001**
	Unilateral	21.84 ± 1.44	19.52 ± 20.51	*p*_2_ = 0.716
	*p*	*p*_3_ = 0.169	***p***_**3**_ **= 0.004**	

*Note: p* value: *p*_1_ referenced to the difference between preoperative and the last follow‐up in the bilateral group, *p*_2_ referenced to the difference between preoperative and the last follow‐up in the unilateral group, and *p*_3_ referenced to the difference between the bilateral and the unilateral groups. *p* values indicating a significant difference between groups are in bold.

Abbreviations: AOFAS, American orthopedic foot and ankle society; HHS, Harris hip score; KOOS, knee injury and osteoarthritis outcome score; WOMAC, Western Ontario McMaster osteoarthritis index.

### 3.3 Lower Limb Alignment of Unilateral Patients

Table 3 presents the results of radiographic measurement for patients who underwent unilateral THA. Preoperatively, significant differences were observed in all the knee parameters and part of ankle parameters (mLDTA and PACO) between the ipsilateral lower limb and control group, indicating valgus knee deformity and varus ankle deformity before THA. For the contralateral lower limb, significant differences were observed in part of the knee parameters (MAD, aTFA, and HKA) between DDH and control groups, indicating slight valgus knee deformity. There was no difference in all three ankle parameters between the contralateral lower limb of the DDH group and the control group.

**TABLE 3:** Parameters of X‐rays of unilateral patients.

		Ipsilateral (mean ± SD)					Contralateral (mean ± SD)				
		Before THA	Immediately after THA	Last follow‐up	*p*_1_	*p*_2_	Before THA	Immediately after THA	Last follow‐up	*P*_1_	*P*_2_
Knee parameters											
MAD (mm)	Unilateral	12.66 ± 6.18	9.05 ± 4.31	7.64 ± 3.53	*p*_1_ = 0.055	***p***_**2**_ **< 0.001**	9.83 ± 5.66	9.68 ± 5.66	5.65 ± 4.84	*p*_1_ = 0.883	***p***_**2**_ **< 0.001**
	Control	3.97 ± 2.90					3.97 ± 2.90				
	*p*_3_	***p***_**3**_ **< 0.001**	***p***_**3**_ **< 0.001**	***p***_**3**_ **< 0.001**			***p***_**3**_ **< 0.001**	***p***_**3**_ **< 0.001**	*p*_3_ = 0.164		
aTFA (°)	Unilateral	172.16 ± 2.37	174.28 ± 2.83	174.24 ± 1.98	***p***_***1***_ **= 0.007**	***p***_**2**_ **= 0.002**	174.91 ± 2.75	175.76 ± 2.94	176.69 ± 3.29	***p***_**1**_ **= 0.049**	***p***_**2**_ **= 0.002**
	Control	176.81 ± 2.25					176.81 ± 2.25				
	*p*_3_	***p***_**3**_ **< 0.001**	***p***_**3**_ **= 0.002**	***p***_**3**_ **< 0.001**			***p***_**3**_ **= 0.016**	*p*_3_ = 0.188	*p*_3_ = 0.883		
HKA (°)	Unilateral	176.86 ± 2.07	176.49 ± 1.96	177.57 ± 1.12	*p*_1_ = 0.570	*p*_2_ = 0.082	176.58 ± 1.66	177.04 ± 1.38	178.22 ± 1.76	*p*_1_ = 0.115	*p*_*2*_ = 0.078
	Control	178.38 ± 0.83					178.38 ± 0.83				
	*p*_3_	***p***_**3**_ **= 0.003**	***p***_**3**_ **< 0.001**	***p***_**3**_ **= 0.009**			***p***_**3**_ **< 0.001**	***p***_**3**_ **< 0.001**	*p*_3_ = 0.302		
mLDFA (°)	Unilateral	82.83 ± 2.72	85.15 ± 2.01	85.24 ± 2.56	***p***_**1**_ **< 0.001**	***p***_**2**_ **< 0.001**	90.45 ± 4.99	85.49 ± 15.18	90.77 ± 2.38	*p*_1_ = 0.092	*p*_2_ = 0.755
	Control	90.16 ± 1.53					90.16 ± 1.53				
	*p*_3_	***p***_**3**_ **< 0.001**	***p***_**3**_ **< 0.001**	***p***_**3**_ **< 0.001**			*p*_3_ = 0.775	*p*_3_ = 0.159	*p*_3_ = 0.314		
mMPTA (°)	Unilateral	91.50 ± 3.18	90.76 ± 3.01	89.57 ± 2.55	*p*_1_ = 0.234	*p*_**2**_ **= 0.012**	88.77 ± 0.51	87.23 ± 2.65	88.28 ± 1.98	***p***_**1**_ **= 0.019**	*p*_*2*_ = 0.251
	Control	88.52 ± 1.67					88.52 ± 1.67				
	*p*_3_	*p*_**3**_ **< 0.001**	***p***_**3**_ **= 0.004**	*p*_3_ = 0.115			*p*_3_ = 0.694	*p*_3_ = 0.060	*p*_3_ = 0.665		
HMFC (mm)	Unilateral	15.15 ± 1.91	14.51 ± 2.85	13.69 ± 2.83	*p*_1_ = 0.332	***p***_**2**_ **< 0.034**	11.99 ± 3.10	11.43 ± 2.71	10.87 ± 2.24	*p*_1_ = 0.437	*p*_2_ = 0.116
	Control	11.51 ± 1.39					11.51 ± 1.39				
	*p*_3_	***p***_**3**_ **< 0.001**	***p***_**3**_ **< 0.001**	***p***_**3**_ **= 0.002**			*p*_3_ = 0.510	*p*_3_ = 0.906	*p*_3_ = 0.264		
HLFC (mm)	Unilateral	4.29 ± 1.75	4.75 ± 3.41	5.01 ± 2.89	*p*_1_ = 0.610	*p*_2_ = 0.268	5.89 ± 2.17	5.35 ± 1.58	5.94 ± 2.55	*p*_1_ = 0.349	*p*_*2*_ = 0.944
	Control	4.87 ± 1.47					4.87 ± 1.47				
	*p*_3_	*p*_3_ = 0.240	*p*_3_ = 0.882	*p*_3_ = 0.845			*p*_3_ = 0.076	*p*_3_ = 0.302	*p*_3_ = 0.96		
FO (mm)	Unilateral	27.25 ± 6.27	29.68 ± 4.83	31.18 ± 4.48	*p*_1_ = 0.211	***p***_**2**_ **= 0.036**	42.45 ± 5.27	42.63 ± 5.52	42.25 ± 6.21	*p*_1_ = 0.831	*p*_*2*_ = 0.779
	Control	45.63 ± 5.27					45.63 ± 5.27				
	*p*_3_	***p***_**3**_ **< 0.001**	***p***_**3**_ **< 0.001**	***p***_**3**_ **< 0.001**			*p*_3_ = 0.052	*p*_3_ = 0.072	*p*_3_ = 0.058		
Ankle parameters											
mLDTA (°)	Unilateral	87.58 ± 2.35	89.05 ± 4.02	90.11 ± 4.32	*p*_1_ = 0.139	***p***_**2**_ **= 0.011**	91.10 ± 2.07	92.06 ± 2.76	90.55 ± 2.16	*p*_1_ = 0.215	*p*_*2*_ = 0.295
	Control	91.52 ± 1.93					91.52 ± 1.93				
	*p*_3_	***p***_**3**_ **< 0.001**	*p*_3_ = 0.14	*p*_3_ = 0.174			*p*_3_ = 0.489	*p*_3_ = 0.453	*p*_3_ = 0.123		
FACO (mm)	Unilateral	5.45 ± 2.66	4.72 ± 2.97	4.25 ± 1.17	*p*_1_ = 0.418	***p***_**2**_ **= 0.032**	1.41 ± 0.78	2.40 ± 1.51	1.91 ± 1.83	***p***_**1**_ **= 0.024**	*p*_*2*_ = 0.294
	Control	1.96 ± 1.27					1.96 ± 1.27				
	*p*_3_	***p***_**3**_ **< 0.001**	***p***_**3**_ **< 0.001**	***p***_**3**_ **< 0.001**			*p*_3_ = 0.093	*p*_3_ = 0.295	*p*_3_ = 0.917		
TT (°)	Unilateral	1.43 ± 0.97	0.89 ± 0.74	1.23 ± 0.58	***p***_**1**_ **= 0.026**	*p*_2_ = 0.408	0.93 ± 0.66	1.10 ± 0.79	1.00 ± 1.08	*p*_1_ = 0.332	*p*_2_ = 0.820
	Control	0.94 ± 0.65					0.94 ± 0.65				
	*p*_3_	*p*_3_ = 0.058	*p*_3_ = 0.812	*p*_3_ = 0.131			*p*_3_ = 0.938	*p*_3_ = 0.480	*p*_3_ = 0.831		

*Note: p* value: *p*_1_ referenced to the difference between preoperative and immediately postoperative, *p*_2_ referenced to the difference between preoperative and the last follow‐up, and *p*_3_ referenced to the difference between DDH and the control group. *p* values indicating a significant difference between groups are in bold.

Abbreviations: aTFA, anatomical tibiofemoral angle; FACO, fibula axis – calcaneal overlap; FO, femoral offset; HKA, hip‐knee‐ankle angle; HLFC, height of lateral femoral condyle; HMFC, height of medial femoral condyle; MAD, mechanical axis deviation; mLDFA, mechanical lateral distal femoral angle; mLDTA, mechanical lateral distal tibial angle; mMPTA, mechanical medial proximal tibial angle; TT, talar tilt.

At the end of follow‐up, six knee (MAD, aTFA, mLDFA, mMPTA, HMFC, and FO) and two ankle parameters (mLDFA and FACO) of the ipsilateral lower limb were significantly changed compared to preoperative values, indicating that the valgus knee deformity and varus ankle deformity were both gradually improved after THA. However, there were significant differences between DDH and control groups in terms of 6 knee (MAD, aTFA, HKA, mLDFA, HMFC, and FO) and one ankle (FACO) parameter, indicating that ipsilateral knee valgus and ankle varus deformity existed after THA. For the contralateral lower limb, at the end of follow‐up, no significant differences were found between DDH and control groups in all the parameters of the knee joint and ankle joint, indicating that contralateral lower alignment was fully corrected after THA.

### 3.4 Lower Limb Alignment of Bilateral Patients

Table 4 shows the radiographic measurements for patients who underwent bilateral THA. Significant differences were observed in most knee and ankle parameters between the DDH and control groups, indicating valgus knee deformity and varus ankle deformity in bilateral DDH patients.

**TABLE 4:** Parameters of X‐rays of bilateral patients.

		Before THA (mean ± SD)	Immediately after THA (mean ± SD)	Last follow‐up (mean ± SD)	*p*_1_	*p*_2_
Knee parameters						
MAD (mm)	Bilateral	11.13 ± 6.50	9.49 ± 5.86	9.23 ± 5.98	***p***_**1**_ **< 0.001**	***p***_**2**_ **< 0.001**
	Control	2.95 ± 3.25				
	*p*_3_	***p***_**3**_ **= 0.003**	***p***_**3**_ **< 0.001**	***p***_**3**_ **< 0.001**		
aTFA (°)	Bilateral	173.99 ± 3.11	175.21 ± 2.31	174.14 ± 3.32	***p***_**1**_ **< 0.001**	***p***_**2**_ **< 0.001**
	Control	178.39 ± 2.04				
	*p*_3_	***p***_**3**_ **< 0.001**	***p***_**3**_ **< 0.001**	***p***_**3**_ **< 0.001**		
HKA (°)	Bilateral	176.98 ± 2.06	177.54 ± 2.11	177.29 ± 2.14	***p***_**1**_ **< 0.001**	***p***_**2**_ **< 0.001**
	Control	178.39 ± 1.26				
	*p*_3_	***p***_**3**_ **< 0.001**	***p***_**3**_ **< 0.001**	***p***_**3**_ **< 0.001**		
mLDFA (°)	Bilateral	86.34 ± 3.19	88.03 ± 3.28	88.01 ± 3.12	***p***_**1**_ **< 0.001**	***p***_***2***_ **< 0.001**
	Control	89.13 ± 1.94				
	*p*_3_	***p***_**3**_ **< 0.001**	***p***_**3**_ **< 0.001**	***p***_**3**_ **< 0.001**		
mMPTA (°)	Bilateral	90.72 ± 4.84	89.91 ± 2.19	90.07 ± 2.47	*p*_1_ = 0.107	*p*_2_ = 0.610
	Control	88.17 ± 1.79				
	*p*_3_	*p*_3_ = 0.636	***p***_**3**_ **= 0.003**	*p*_3_ = 0.193		
HMFC (mm)	Bilateral	14.85 ± 1.38	13.61 ± 1.70	13.54 ± 1.83	***p***_**1**_ **< 0.001**	***p***_**2**_ **< 0.001**
	Control	12.35 ± 1.44				
	*p*_3_	***p***_**3**_ **< 0.001**	***p***_**3**_ **< 0.001**	***p***_**3**_ **< 0.001**		
HLFC (mm)	Bilateral	3.94 ± 1.47	4.10 ± 1.24	4.34 ± 1.84	***p***_**1**_ **< 0.001**	***p***_**2**_ **< 0.001**
	Control	5.76 ± 1.41				
	*p*_3_	***p***_**3**_ **< 0.001**	***p***_**3**_ **< 0.001**	***p***_**3**_ **< 0.001**		
FO (mm)	Bilateral	31.09 ± 5.25	37.85 ± 3.38	37.75 ± 3.79	***p***_**1**_ **< 0.001**	***p***_**2**_ **< 0.001**
	Control	49.36 ± 4.22				
	*p*_3_	***p***_**3**_ **< 0.001**	***p***_**3**_ **< 0.001**	***p***_**3**_ **< 0.001**		
Ankle parameters						
mLDTA (°)	Bilateral	87.92 ± 2.77	89.21 ± 3.59	89.73 ± 3.30	*p*_1_ = 0.423	*p*_2_ = 0.922
	Control	91.06 ± 1.82				
	*p*_3_	***p***_**3**_ **< 0.001**	***p***_**3**_ **= 0.002**	***p***_**3**_ **= 0.005**		
FACO (mm)	Bilateral	6.83 ± 2.82	5.12 ± 2.26	4.60 ± 2.88	***p***_**1**_ **< 0.001**	***p***_**2**_ **< 0.001**
	Control	2.49 ± 1.82				
	*p*_3_	***p***_**3**_ **< 0.001**	***p***_**3**_ **< 0.001**	***p***_**3**_ **< 0.001**		
TT (°)	Bilateral	1.49 ± 0.99	1.35 ± 0.86	1.33 ± 0.95	*p*_1_ = 0.235	*p*_2_ = 0.960
	Control	1.01 ± 0.73				
	*p*_3_	*p*_3_ = 0.248	*p*_3_ = 0.186	*p*_3_ = 0.996		

*Note: p* value: *p*_1_ referenced to the difference between preoperative and immediately postoperative, *p*_2_ referenced to the difference between preoperative and the last follow‐up, and *p*_3_ referenced to the difference between DDH and control group. *p* values indicating a significant difference between groups are in bold.

Abbreviations: aTFA, anatomical tibiofemoral angle; FACO, fibula axis–calcaneal overlap; FO, femoral offset; HKA, hip‐knee‐ankle angle; HLFC, height of lateral femoral condyle; HMFC, height of medial femoral condyle; MAD, mechanical axis deviation; mLDFA, mechanical lateral distal femoral angle; mLDTA, mechanical lateral distal tibial angle; mMPTA, mechanical medial proximal tibial angle; TT, talar tilt.

At the last follow‐up, significant improvements from preoperative values were found in four knee parameters (MAD, mLDFA, HMFC, and FO; all *p* < 0.001) and one ankle parameter (FACO; *p* < 0.001), suggesting partial correction of valgus knee and varus ankle deformities after THA. However, no significant changes were observed in mMPTA (*p* = 0.610), mLDTA (*p* = 0.922), or TT (*p* = 0.960) between preoperative and final follow‐up measurements.

Compared with the control group, DDH patients showed significant differences at the last follow‐up in MAD (*p* < 0.001), aTFA (*p* < 0.001), HKA (*p* < 0.001), HMFC (*p* < 0.001), HLFC (*p* < 0.001), FO (*p* < 0.001), and FACO (*p* < 0.001), while no significant differences were found in mMPTA (*p* = 0.193), mLDTA (*p* = 0.005), or TT (*p* = 0.996). These findings indicate that complete correction of knee valgus and ankle varus deformities was not achieved in DDH patients at an average follow‐up of 10.2 years after THA.

### 3.5 3D Gait Analysis Outcomes

Table 5 shows the results of the 3D gait analysis. Regarding spatiotemporal and kinematic parameters, the stride length of both DDH groups was significantly shorter compared to the control group (Unilateral: *p* = 0.022, Bilateral: *p* < 0.001). However, only the bilateral DDH group exhibited a significantly reduced walking speed compared to the control (*p* < 0.001). Both DDH groups showed a significantly decreased hip flexion‐extension range of motion compared to the control group (*p* < 0.001). In contrast, the ankle plantarflexion‐dorsiflexion range of motion was significantly greater in both DDH groups compared to the control (*p* < 0.001). The bilateral DDH group showed a significantly increased knee flexion‐extension range of motion compared to the control group (*p* = 0.012).

**TABLE 5:** Parameters of gait analysis at the last follow‐up (mean ± SD).

Parameters	Controls group	Unilateral DDH group	Bilateral DDH group	*p*		
				Control vs. Unilateral	Control vs. Bilateral	Bilateral vs. Unilateral
Spatiotemporal parameters						
Speed (m/s)	1.21 ± 0.10	1.02 ± 0.19	0.88 ± 0.13	*p* = 0.201	***P* < 0.001**	*p* = 0.321
Stance time (s)	0.62 ± 0.03	0.67 ± 0.07	0.68 ± 0.07	*p* = 0.317	***p* = 0.012**	*p* = 0.954
Gait cycle time (s)	1.02 ± 0.05	1.11 ± 0.08	1.10 ± 0.09	*p* = 0.073	***p* = 0.018**	*p* = 0.948
Step length (m)	1.21 ± 0.11	1.10 ± 0.13	0.95 ± 0.10	***p* = 0.022**	***p* < 0.001**	***p* = 0.004**
Kinematics						
Hip flexion range of motion (°)	40.9 ± 4.7	27.2 ± 3.2	23.9 ± 5.3	***p* < 0.001**	***p* < 0.001**	*p* = 0.203
Hip adduction range of motion (°)	11.7 ± 1.4	9.3 ± 2.5	8.1 ± 2.6	***p* = 0.024**	***p* < 0.001**	*p* = 0.345
Knee flexion range of motion (°)	38.6 ± 3.4	40.8 ± 5.6	44.4 ± 7.1	*p* = 0.625	***p* = 0.012**	*p* = 0.402
Ankle plantarflexion range of motion (°)	20.7 ± 3.4	26.4 ± 3.2	26.0 ± 4.4	***p* < 0.001**	***p* < 0.001**	*p* = 0.956
Kinetics						
Peak hip joint contact force (BW)	4.18 ± 0.77	2.88 ± 0.67	2.69 ± 0.32	***p* = 0.025**	***p* < 0.001**	*p* = 0.832
Peak knee joint contact force (BW)	4.03 ± 0.77	2.76 ± 0.48	2.69 ± 0.66	***p* = 0.009**	***p* = 0.002**	*p* = 0.701
Peak medial knee contact force (BW)	2.98 ± 0.57	1.78 ± 0.29	1.57 ± 0.65	***p* < 0.001**	***p* < 0.001**	*p* = 0.603
Peak lateral knee contact force (BW)	1.42 ± 0.27	1.43 ± 0.35	1.30 ± 0.49	*p* = 0.999	*p* = 0.135	*p* = 0.384
Lateral contact force ratio (%)	35.6 ± 5.2	51.9 ± 9.3	49.2 ± 15.6	***p* = 0.047**	***p* < 0.001**	*p* = 0.402
Peak ankle joint contact force (BW)	5.93 ± 0.45	4.97 ± 0.45	5.21 ± 0.54	***p* < 0.001**	***p* < 0.001**	*p* = 0.461
Peak hip flexion moment (N·m/kg)	0.85 ± 0.15	0.63 ± 0.17	0.56 ± 0.13	***p* < 0.001**	***p* < 0.001**	*p* = 0.483
Peak hip adduction moment (N·m/kg)	0.77 ± 0.10	0.48 ± 0.14	0.54 ± 0.13	***p* < 0.001**	***p* < 0.001**	*p* = 0.498
Peak knee flexion moment (N·m/kg)	0.51 ± 0.13	0.32 ± 0.07	0.30 ± 0.08	***p* < 0.001**	***p* < 0.001**	*p* = 0.822
Peak knee adduction moment (N·m/kg)	0.31 ± 0.07	0.08 ± 0.06	0.10 ± 0.09	***p* < 0.001**	***p* < 0.001**	*p* = 0.989
Peak ankle plantarflexion moment (N·m/kg)	1.38 ± 0.12	1.18 ± 0.14	1.14 ± 0.10	***p* < 0.001**	***p* < 0.001**	*p* = 0.721

*Note: p* values indicating a significant difference between groups are in bold.

Regarding kinetic parameters, both DDH groups showed significantly reduced peak knee joint contact force (Unilateral: *p* = 0.009, Bilateral: *p* = 0.002), medial knee joint contact force (*p* < 0.001), and knee adduction moment (*p* < 0.001) compared to the control group. However, no significant differences were observed in peak lateral knee joint contact force between the DDH groups and the control. Furthermore, both DDH groups exhibited significantly higher lateral contact force ratios compared to the control group (Unilateral: *p* = 0.047, Bilateral: *p* = 0.001).

### 3.6 Plantar Force Outcomes

The results of the plantar force analysis are presented in Table 6. The parameters of HM, HL, and FM in the unilateral DDH group were significantly lower than those in the control group (*p* < 0.001), except for MF, FI, FL, and T. Significantly differences were found between the bilateral DDH and control groups in terms of HL and FM parameters (*p* < 0.001). No significant differences were found between the two DDH groups in all the parameters of plantar force examination.

**TABLE 6:** Peak plantar force at the last follow‐up.

Parameters	Controls group	Unilateral DDH group	Bilateral DDH group	*p*		
				Control vs. Unilateral	Control vs. Bilateral	Bilateral vs. Unilateral
HM (medial heel) (BW)	0.34 ± 0.05	0.26 ± 0.04	0.30 ± 0.05	***p* < 0.001**	*p* = 0.056	*p* = 0.158
HL (lateral heel) (BW)	0.36 ± 0.06	0.24 ± 0.05	0.25 ± 0.05	***p* < 0.001**	***p* < 0.001**	*p* = 0.982
MF (midfoot) (BW)	0.19 ± 0.09	0.19 ± 0.06	0.19 ± 0.07	*p* = 0.997	*p* = 0.952	*p* = 0.985
FM (medial forefoot) (BW)	0.29 ± 0.06	0.21 ± 0.05	0.17 ± 0.05	***p* < 0.001**	***p* < 0.001**	*p* = 0.077
FI (intermediate forefoot) (BW)	0.36 ± 0.08	0.39 ± 0.06	0.35 ± 0.04	*p* = 0.631	*p* = 0.724	*p* = 0.276
FL (lateral forefoot) (BW)	0.13 ± 0.04	0.12 ± 0.04	0.15 ± 0.04	*p* = 0.804	*p* = 0.515	*p* = 0.283
T (toe) (BW)	0.25 ± 0.05	0.22 ± 0.05	0.24 ± 0.08	*p* = 0.341	*p* = 0.881	*p* = 0.558

*Note: p* values indicating a significant difference between groups are in bold.

### 3.7 Complications and Prosthesis Survival Rate

Solid bone union in all the enrolled patients was archived at the osteotomy site without any complication. Two patients (2 hips) experienced postoperative dislocation and were treated with closed reduction without recurrence. The postoperative limp was moderate in 3 cases and slight in 5 cases; others recovered fully. Intraoperative fracture occurred in 2 hips, which were treated with cerclage cable and healed without further sequelae. Critically, at the mean follow‐up of 10.2 years, no cases of deep venous thrombosis, periprosthetic infection, aseptic loosening, or osteolysis were observed. Consequently, none of the implants required revision surgery, yielding a 100% prosthesis retention rate free from major complications.

## 4 Discussion

### 4.1 Long‐Term Efficacy of THA in Improving Function and Alignment

The long‐term postoperative changes in lower limb axial alignment for Crowe IV DDH patients after THA remain unclear. Our present study revealed persistent abnormal lower limb alignment in these patients after THA, which also affected their gait and plantar force. Our radiographic results demonstrated that while THA significantly improved several key knee alignment parameters (MAD, mLDFA, HMFC, and FO), complete normalization was not achieved, and ankle alignment parameters showed variable responses to surgical intervention. To our knowledge, this is the first study to investigate whether THA can improve lower limb alignment in Crowe IV DDH patients with a mean follow‐up of over 10 years.

DDH significantly impairs lower limb function, especially in highly dislocated Crowe IV patients [21]. Previous literature extensively reported valgus lower limb alignment after THA in patients with high dislocation in DDH [6, 7, 22]. This alignment deviation has been associated with long‐term postoperative pain and the development of knee arthritis [23, 24]. Our previous studies predominantly demonstrated a valgus knee position in patients with high dislocation both preoperatively and within the initial 1–2 years following THA [7]. In this study, we further revealed that THA produced differential effects on various knee alignment parameters in Crowe IV DDH patients, and residual abnormalities persisted throughout 10 years of postoperative follow‐up. Additionally, limited research has explored the impact of high dislocation in DDH on the ankle joint. Our current study suggested that the prolonged lower limb imbalance resulting from DDH could influence ankle joint alignment. Specifically, our study found variable correction patterns in ankle parameters, with FACO showing improvement while mLDTA and TT remained largely unchanged throughout the extended postoperative period.

### 4.2 Comprehensive Biomechanical Outcomes: Gait, Kinetics, and Plantar Pressure

In our current study, we enrolled Crowe IV DDH patients who underwent unilateral and bilateral THA and enabled a comparison between the two DDH groups and healthy individuals. Regarding joint function and quality of life scores, most scales showed significant improvement after THA. However, the WOMAC score did not significantly improve in the unilateral group. We believe that the abnormal development of the unilateral hip joint may lead to a greater imbalance between the ipsilateral and contralateral sides compared to the bilateral group. This imbalance affected the restoration of soft tissues after THA, as reflected in the WOMAC score. Regarding the changes in lower limb force, most parameters of the ipsilateral hip joints in both groups significantly improved but remained inferior to the control group. Interestingly, our study showed that the preoperative valgus deformity of the contralateral knee was fully normal after THA for the unilateral DDH group. This finding suggested that THA not only benefits the affected limb's alignment and function but also plays a crucial role in protecting the contralateral limb.

Walking speed serves as a comprehensive indicator of walking capability. According to our research, DDH patients exhibited lower walking speeds than healthy controls 10 years after surgery, especially in bilateral DDH patients, indicating reduced walking capability. Lower walking speed often accompanies a smaller joint range of motion [25]. In our study, the reduced walking speed in DDH patients resulted in decreased hip flexion‐extension range of motion without a corresponding decrease in knee and ankle range of motion. Instead, DDH patients demonstrated larger knee flexion‐extension and ankle plantarflexion‐dorsiflexion ranges of motion compared to healthy controls. These findings suggested that DDH patients compensated for restricted hip range of motion by utilizing increased knee and ankle joint ranges of motion during walking.

Additionally, the kinetic data presented in Table 5 suggest potential compensatory mechanisms occurring on the contralateral side in individuals with unilateral DDH. The results indicate that peak joint contact forces and moments at the affected hip and knee were markedly reduced compared to those observed in healthy controls. This diminished loading on the affected limb likely stems from a prolonged adaptation strategy involving partial weight avoidance, leading patients to increasingly depend on the unaffected limb during ambulation. While this research did not explicitly analyze load distribution patterns, the observed asymmetry aligns with previously reported compensation strategies in other unilateral musculoskeletal conditions [26]. Importantly, even after THA restores the lower limb alignment on the affected side, as confirmed by imaging, the non‐operated limb may continue to play a dominant role in generating propulsive force and maintaining balance during gait. Further investigations should incorporate comprehensive bilateral kinematic and kinetic analyses to better quantify these adaptive responses and evaluate their influence on long‐term functional recovery and clinical prognosis.

The medial and lateral contact forces within the knee joint closely correlate with lower limb alignment [27, 28]. Previous studies have shown that valgus lower limb alignment shifts knee joint contact force from the medial compartment to the lateral compartment [28] In our study, DDH patients exhibited a higher peak lateral contact force compared to healthy controls, indicating a shift of loads from the lateral compartment to the medial compartment during walking. This finding was consistent with our observation of valgus lower limb alignment in DDH patients. Plantar forces reflect force distribution across different foot regions during walking. In our study, DDH patients showed reduced peak medial forefoot force compared to healthy controls, likely due to lower walking speeds [29] However, peak lateral forefoot force remained unchanged. This finding suggested that DDH patients tend to shift plantar force from the medial forefoot to the lateral forefoot, which also supported the presence of valgus lower limb alignment in DDH patients.

### 4.3 Clinical Implications and Management Strategies Based on Long‐Term Findings

The persistent biomechanical abnormalities observed in our study carry important clinical implications for the long‐term management of Crowe IV DDH patients after THA. The combination of valgus knee alignment and elevated lateral compartment contact forces is a well‐established risk factor for the development and progression of lateral knee osteoarthritis [30]. This altered loading environment may accelerate cartilage wear in the lateral compartment, potentially explaining why some patients continue to experience knee pain despite a radiologically successful THA. Similarly, the observed shift in plantar load towards the lateral forefoot suggests an adaptive, albeit potentially pathological, gait pattern. This redistribution may potentially increase the risk of metatarsalgia, stress fractures in the lateral rays, and plantar fasciitis [31]. Therefore, the residual gait and alignment deviations we documented may underlie a spectrum of secondary musculoskeletal complaints, highlighting the need for long‐term surveillance and targeted interventions aimed at mitigating these risks.

Based on our decade‐long observations, we suggest several clinical management strategies. First, postoperative rehabilitation should be emphasized and prolonged, building upon standard physiotherapy to specifically target strengthening of the hip abductors and gluteal muscles, as well as gait re‐education to promote symmetrical weight‐bearing and movement patterns. Second, a long‐term monitoring protocol should be established that extends beyond typical implant surveillance, incorporating regular clinical assessment for knee and ankle symptoms and consideration of periodic full‐limb alignment radiographs to monitor for progressive valgus or joint space narrowing. Finally, patient education is crucial. Patients should be counseled that complete biomechanical normalization may not be achievable, and that ongoing gait deviations are common. Guidance on activity modification and weight management is essential to reduce cumulative joint stress.

### 4.4 Etiology of Persistent Abnormalities and Study Limitations

A critical question arising from our findings is why these biomechanical and alignment abnormalities persist even a decade after THA. We postulate that this incomplete correction is multifactorial, rooted in the profound and chronic nature of Crowe IV DDH. On the one hand, irreversible bony morphological changes are a primary contributor. The hypoplastic acetabulum and the altered geometry of the proximal femur, despite reconstruction, can result in a permanent alteration of the hip's center of rotation and functional lever arms, thereby influencing the entire kinematic chain of the lower limb [32, 33]. On the other hand, soft tissue adaptations play a crucial role. Long‐standing superior dislocation leads to contractures of the abductor and iliotibial band complexes, capsular laxity inferiorly, and muscular imbalances (e.g., gluteus medius weakness). THA may not fully normalize these soft tissue tensions and muscle functions, leading to persistent gait deviations like Trendelenburg gait or abductor lurch [34].

The present study intended to characterize the overall long‐term biomechanical outcomes following THA in patients with Crowe IV DDH. It should be noted that our patient cohort included a mix of bearing surfaces, such as ceramic‐on‐ceramic, metal‐on polyethylene, and different stem designs. This variation reflects the real‐world clinical practice over the decade‐long recruitment period, where prosthesis selection was tailored to individual patient factors like age, activity level, and economic conditions. While this diversity enhances the generalizability of our findings across broad clinical settings, it also makes it methodologically challenging within the present analysis to dissect the isolated impact of specific prosthesis designs on alignment, gait, and functional outcomes with sufficient statistical power while controlling for the aforementioned confounders. Therefore, the residual biomechanical abnormalities observed in our study represent a common challenge that persists despite various successful surgical strategies. Future investigations utilizing prospective registry data or studies specifically designed to compare implant types will be crucial to elucidate the role of these important technical factors.

Our study has several limitations that should be considered when interpreting the findings. Firstly, the absence of preoperative gait and plantar pressure data, inherent to our retrospective design, prevents quantification of the functional improvement directly attributable to THA and confines our analysis to depicting the long‐term status. Secondly, the relatively small sample size may limit the statistical power for some subgroup analyses and the generalizability of our results. While we observed an excellent clinical outcome with a 100% prosthesis retention rate free from major complications like revision, aseptic loosening, or infection at a mean of 10.2 years, the limited cohort size necessitates caution in extrapolating this result to broader populations. Thirdly, the majority of our patients were middle‐aged at surgery, raising questions about the generalizability of our findings to younger or older populations with congenital DDH. Furthermore, our assessment of lower limb alignment relied on two‐dimensional radiographs, which, while clinically standard, may not capture three‐dimensional deformities as effectively as CT‐based models. Finally, the diversity in prosthesis designs, although reflective of real‐world surgical practice over a decade, introduces confounding variables that our study was not powered to dissect.

### 4.5 Implications and Future Research

Despite these limitations, the unique 10‐year follow‐up data presented here provide the first comprehensive characterization of the persistent biomechanical and alignment challenges in Crowe IV DDH patients after THA. The compelling finding that no major complications occurred in this complex patient cohort underscores the procedural success and implant resilience achievable with contemporary techniques, even in the presence of persistent biomechanical alterations. These findings offer crucial preliminary evidence and underscore the imperative for future prospective studies with larger cohorts, incorporating comprehensive biomechanical assessments and 3D imaging preoperatively and at multiple postoperative intervals.

## 5 Conclusions

In conclusion, our present study found incomplete postoperative correction of ipsilateral knee valgus and ankle varus deformity in Crowe IV DDH patients, while full correction was achieved contralaterally. Persistent impairments in gait kinematics and plantar force were also noted.

## Author Contributions

Study design and manuscript writing (H.C., J.W., S.T.L.P., Y.Z., J.Y.); Patient follow‐up (H.C., J.W., Y.N.); Anteroposterior projection full‐length standing radiograph and image processing (S.Y., Y.L.); Surgical process (J.Y., S.T.L.P., Y.Z.); Gait analysis (J.W., Y.N.); Data checking (J.Y., Y.Z.).

## Funding

This study was jointly supported by the National Key Research and Development Program of China (No. 2023YFB4606700), Clinical Research Incubation Program of West China Hospital (No. 2024HXFH008), Key Research and Development Program of the Sichuan Science and Technology Department (No. 23ZDYF2641), Cadre Health Research Program of Sichuan Province (No. 2023‐118), and International Science and Technology Cooperation Program of Chengdu Science and Technology Bureau (No. 2023‐GH02‐00075‐HZ).

## Disclosure

The authors have nothing to report.

## Conflicts of Interest

The authors declare no conflicts of interest.

## Supporting information


**Table S1:** The inter‐ and intra‐observer reliability of radiographic measurements.

## Data Availability

The data that support the findings of this study are available on request from the corresponding author. The data are not publicly available due to privacy or ethical restrictions.
