# Supplementary material for: Changes in Lower Limb Axial Alignment, Gait Biomechanics, and Plantar Force in Crowe Type IV Hip Dysplasia After Total Hip Arthroplasty: A Mean Ten‐Year Follow‐Up Retrospective Cohort Study
Source: Orthop Surg. 2026 Feb 6;18(3):459–73. doi: 10.1111/os.70258 (PMC12967602; doi:10.1111/os.70258)
Supplement: Supplementary file 1 — Table S1: The inter‐ and intra‐observer reliability of radiographic measurements. [file OS-18-459-s001.docx]

**Supplementary Table 1. The inter- and intra-observer reliability of radiographic measurements**

|  | inter-observer (ICC, 95% CI) | intra-observer (ICC, 95% CI) |
| --- | --- | --- |
| MAD | 0.93(0.89, 0.98) | 0.98(0.95, 0.99) |
| HKA | 0.89(0.74, 0.95) | 0.95(0.90, 0.98) |
| aTFA | 0.96(0.93, 0.98) | 0.96(0.92, 0.98) |
| mLDFA | 0.89(0.79, 0.95) | 0.90(0.80, 0.95) |
| mMPTA | 0,95(0.88, 0.97) | 0,95(0.90, 0.98) |
| HMFC | 0.97(0.94, 0.98) | 0.98(0.95, 0.99) |
| HLFC | 0.99(0.98, 0.99) | 0.99(0.98, 0.99) |
| FO | 0.95(0.90, 0.98) | 0.96(0.92, 0.98) |
| mLDTA | 0.98(0.96, 0.99) | 0.98(0.96, 0.99) |
| FACO | 0.98(0.95, 0.99) | 0.97(0.94, 0.99) |
| TT | 0.96(0.92, 0.98) | 0.90(0.83, 0.96) |

Abbreviation: MAD, mechanical axis deviation; aTFA, anatomical tibiofemoral angle; HKA, hip-knee-ankle angle; mLDFA, mechanical lateral distal femoral angle; mMPTA, mechanical medial proximal tibial angle; HMFC, height of medial femoral condyle; HLFC, height of lateral femoral condyle; FO, femoral offset; mLDTA, mechanical lateral distal tibial angle; FACO, fibula axis – calcaneal overlap; TT, talar tilt; ICC: Intraclass correlation coefficient.
